# Andrew Charles Petter Sims, MD, FRCPsych (Hon)

**DOI:** 10.1192/bjb.2023.23

**Published:** 2023-10

**Authors:** David Sims

Emeritus Professor of Psychiatry, University of Leeds Medical School, UK

**Figure:**
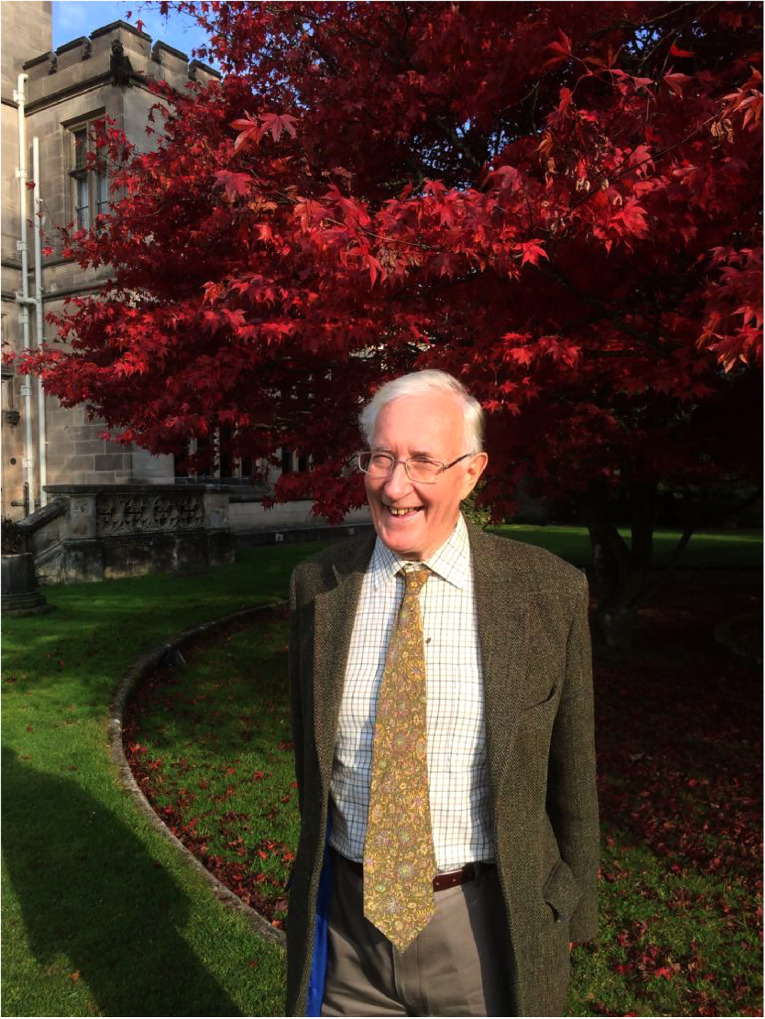

Over many years, Andrew Sims, who died on 14 December 2022 aged 84, published extensively on the beneficial role religious faith could play in improving mental health. A committed, active Christian himself, he wrote and lectured extensively on the links between religious faith and mental disorder, pointing to the evidence that religious beliefs should not be considered delusional. He authored and edited numerous books on the subject, including *Spirituality and Psychiatry* and *Is Faith Delusion?*

He also wrote what is widely considered the standard textbook on descriptive psychopathology, *Symptoms in the Mind*. For generations of trainee psychiatrists, this was the text they consulted when they wanted an authoritative view on the meaning of a psychiatric term. The first edition of this book was published in 1995, with the seventh edition appearing in 2022. Andrew was responsible for the first three editions, with Femi Oyebode authoring subsequent editions.

Andrew played leading roles in the Royal College of Psychiatrists. He was Dean of the College from 1987 to 1990 and President from 1990 to 1993. In 1994 he was the founding editor of the College's journal *Advances in Psychiatric Treatment* (now *BJPsych Advances).* Later he became Chair of the Spirituality Special Interest Group in the College. It was his mission to raise standards of psychiatric practice throughout the world and to this end he lectured all over the world, among other places in India, Pakistan, Nepal, Sri Lanka, Singapore, South Africa, Zambia and the Czech Republic.

Andrew was born in November 1938, the second of four children of Charles Sims and his wife Norah, née Petter. Both were general practitioners in Exeter and leading members of the Christian Brethren. During Andrew's infancy, his father served during the Second World War as a medical officer in the armed forces. His mother carried a very heavy burden while so many doctors were away at the war. Andrew went to school in Exeter and then studied Natural Sciences at Cambridge before going on to Westminster Medical School, London, for clinical studies. He did postgraduate psychiatric training in Manchester and Birmingham. His MD thesis from the University of Cambridge was on prognosis in neurotic disorders.

In 1964 he married Ruth, née Harvey, also a psychiatrist.

He was appointed Professor of Psychiatry in the University of Leeds Medical School in 1979, with an honorary consultant post in psychiatry at St James's University Hospital, Leeds. In addition to his research interests in descriptive psychopathology and post-traumatic stress he developed a clinical academic unit at St James's Hospital which became the first specialist eating disorder unit in Leeds. He retired from both these posts in February 2000, at the age of 62. During his career, he received many honours, including honorary fellowships of the Royal College of Psychiatrists, the College of Physicians and Surgeons Pakistan, the Colleges of Medicine of South Africa and the Association of European Psychiatrists (now the European Psychiatric Association). He was also a life member of the Pakistan Psychiatric Society. He was placed on the Royal College of Psychiatrist's Roll of Honour in 2015.

His role as a Christian leader was recognised in 1995 by the award of Doctor of Medicine from the then Archbishop of Canterbury, George Carey. This rarely awarded Lambeth degree was given ‘in recognition of his services to psychiatry, in particular in promoting the need to evaluate the religious and spiritual experience of patients’.

Andrew was a modest man, his humility being well illustrated when he was the subject of a spontaneous role-play using his own experience of anxiety to teach a group of doctors and nurses with his psychiatrist son David as the cognitive–behavioural therapist. His wife said of him ‘Andrew chose to study psychiatry because he believed it was his calling. He was always full of energy, cheerfulness and compassion. For us as a family, his great sense of humour meant life was always fun with him. But above all we remember his deep love for all of us.’

After his retirement, Andrew and his wife retired to Alveley, near Bridgnorth in Shropshire. They threw themselves into the life of the village church, St Mary's, parts of which date to the 12th century. Ruth was ordained as an Anglican minister, assisting with services there. Andrew continued to teach psychiatry when he could, including alongside David in Nepal working with PRIME (Partnerships in International Medical Education). In Alveley he spent as much time as possible in his garden and enjoying his family as well as singing and attending concerts, while making an annual trip to watch the Test match at Headingly or Edgbaston.

He is survived by Ruth as well as his children David, Mary, John and Ann, and twelve grandchildren.

